# Antibacterial Polyketides from the Marine Alga-Derived Endophitic *Streptomyces sundarbansensis*: A Study on Hydroxypyrone Tautomerism

**DOI:** 10.3390/md11010124

**Published:** 2013-01-10

**Authors:** Ibtissem Djinni, Andrea Defant, Mouloud Kecha, Ines Mancini

**Affiliations:** 1 Laboratory of Applied Microbiology, Faculty of Nature Science and Life, University of Bejaia, Targa Ouzemmour 06000, Algeria; E-Mails: ibtissem.djinni@yahoo.fr (I.D.); kmkmsetif@yahoo.fr (M.K.); 2 Bioorganic Chemistry Laboratory, Department of Physics, University of Trento, via Sommarive 14, I-38123 Povo-Trento, Italy; E-Mail: defant@science.unitn.it

**Keywords:** marine *Streptomyces*, antibacterial activity, phaeochromycins, density functional calculations, hydroxypyrone tautomerism

## Abstract

Polyketide **13** [=2-hydroxy-5-((6-hydroxy-4-oxo-4*H*-pyran-2-yl)methyl)-2-propylchroman-4-one] and three related known compounds **7**, **9** and **11** were obtained and structurally characterized from *Streptomyces sundarbansensis* strain, an endophytic actinomycete isolated from the Algerian marine brown algae *Fucus* sp. Compound **13** was obtained as the major metabolite from optimized culture conditions, by using Agar state fermentation. Due to tautomeric equilibrium, **13** in CD_3_OD solution was able to incorporate five deuterium atoms, as deduced by NMR and ESI-MS/MS analysis. The 2-hydroxy-γ-pyrone form was established for these metabolites based on the comparison of their experimental IR spectra with the DFT calculated ones, for both the corresponding 4-hydroxy-α-pyrone and 2-hydroxy-γ-pyrone forms. During antibacterial evaluation, compound **13** stood out as the most active of the series, showing a selective activity against the gram positive pathogenic methicillin-resistant *S. aureus* (MRSA, MIC = 6 μΜ), with a bacteriostatic effect.

## 1. Introduction

Bacteria proved to be a particularly prolific resource with a surprisingly small group of taxa, which accounted for the vast majority of isolated compounds [1]. Among these taxa, actinomycetes are the most economically and biotechnologically valuable prokaryotes [2], responsible for the production of about half of the discovered bioactive secondary metabolites, including antitumor and immunosuppressive agents and antibiotics [3]. In particular, the genus *Streptomyces* accounts for about 80% of the actinomycete natural products reported to date [4]. Considerable attention is currently focused on the study of new actinomycetes coming from poorly researched habitats, with the aim of finding new bioactive products which are useful for biotechnological access to new potential drugs [5,6]. It has also been suggested that metabolites obtained from algae and invertebrates such as sponges, mollusks and tunicates are actually produced by microorganisms [7,8], hence marine organism-associated actinobacteria are attracting increasing interest as potential sources of natural products.

**Figure 1:**
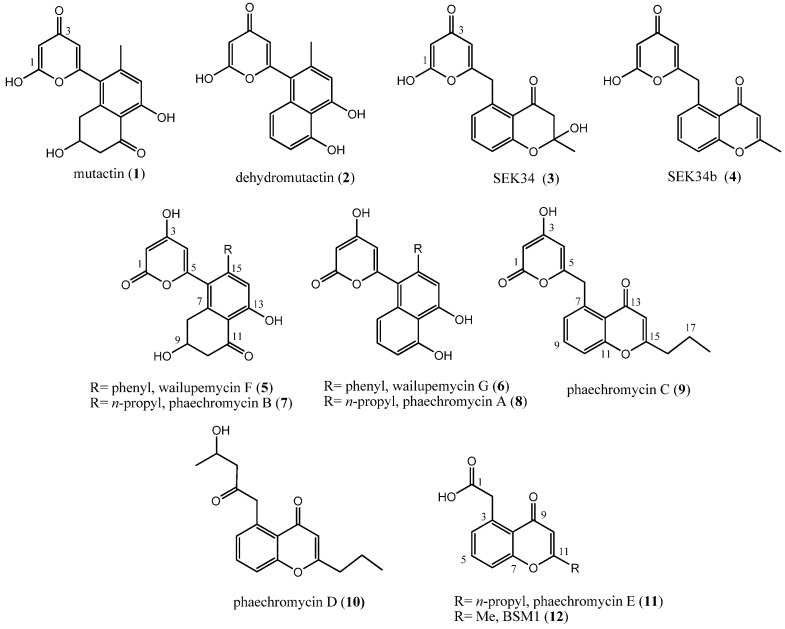
Molecular structures of some polyketides. Arbitrary numbering is for convenience.

Polyketides represent a highly diverse structural class of products found also in bacteria, demonstrating varied biological functions [9]. Over the past decades, the isolation and structural characterization of shunt products from *act* mutants of *Streptomyces coelicolor* derived by classical mutagenesis led to the elucidation of several compounds, which typically show the presence of: (i) a methyl group, as in mutactin (**1**), dehydrated mutactin (**2**), SEK34 (**3**) and its dehydrated SEK34b (**4**) [10,11,12]; (ii) a phenyl group, in wailupemycin F (**5**) and G (**6**) isolated using mutational analysis [13]; (iii) a propyl chain in metabolites recently isolated from an actinomycete strain *Streptomyces phaeochromogenes* LL-P018, phaeochromycin A–E (**7**–**11**) [14]. A phaeochromycin E analogue, BSM1 (**12**) bearing methyl instead of the propyl group has also been reported [15] (Figure 1).

The aim of this work is the isolation of metabolites responsible for the antibacterial activities observed in the crude extract from a marine-derived *Streptomyces* sp., isolated from the algae *Fucus* sp., collected along the Bejaia coastline in Algeria. Pure compounds **7**, **9**, **11** and **13** were structurally characterized and their activities against five pathogenic bacteria were then evaluated. We also report on the tautomerism involved in the hydroxypyrone unit which is present in structures **7**, **9** and **13**, and we discuss a method capable of establishing the α- or γ-forms based on a comparison between experimental and DFT calculated IR spectra. 

## 2. Results and Discussion

### 2.1. Production and Structural Characterization of Metabolites

A pure strain *Streptomyces* sp. WR1L1S8 was isolated from the inner tissue of the brown marine algae *Fucus* sp. collected along Bejaia coastline, Algeria and revealed 100% 16S rRNA genes sequence similarity to *Streptomyces sundarbansensis* MS1/7^T^. The representative specie was recently isolated from sediments of the Sundarbans mangrove forest in India, and reported to produce 2-allyloxyphenol [16].

**Figure 2:**
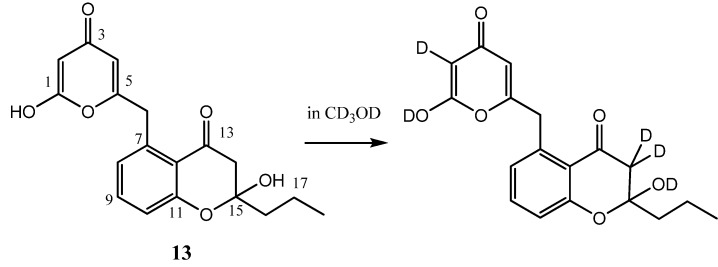
Molecular structure of compound **13** and its derivative by incorporation of deuterium atoms. Numbering is for convenience.

A preliminary study was carried out in order to establish the influence of culture conditions on metabolites production of the strain WR1L1S8, by selecting a process of optimal nutrient medium and fermentation. According to the results, Starch Casein Agar (SCA) supplemented with 50% seawater was the best medium. Moreover, the Agar Surface Fermentation (ASF) process performed on SCA medium of the studied strain permitted a better growth inhibition of the test germs than the submerged fermentation method (SbF). The crude ethyl acetate extract of the strain culture showed promising antibacterial activities. A silica gel chromatography allowed isolation of compounds **7**, **9**, **11** (Figure 1) and **13** (Figure 2), in pure forms as seen by high-performance liquid chromatography (HPLC) analysis. The online HPLC-ESIMS injection of the ethyl acetate extract showed the presence of **13** as a major compound and **7**, **9** and **11**.

The presence in ESI (+)-MS spectrum of the signal at *m/z* 353 attributable to [M + Na]^+^ and in ESI (−)-MS at *m/z* 329 corresponding to [M − H]^−^, suggested the molecular mass of compound **13 **(Figure 2). Electron impact (EI)-MS spectrum showed an intense peak at *m/z* 312 corresponding to the loss of a water molecule in agreement with the ESI-MS fragmentation; high resolution experiment on *m/z* 312 gave the molecular composition C_18_H_16_O_5_.

The downfield region in ^1^H NMR spectrum recorded in CDCl_3_ exhibited three signals, each for one proton, at δ_H_ 7.39 (t, *J* = 8.2 Hz), 6.97 (s) and 6.91 (d, *J* = 8.2 Hz) assigned as belonging in a contiguous position of the same aromatic ring, and two singlets at δ_H_ 5.75 and 5.33, that the HMBC correlations observed for the latter one allowed to assign at H-4 and H-2, respectively (Table 1). A methylene group was detected by the presence of two doublets with the same geminal coupling constant (*J* = 16.4 Hz) at δ_H_ 4.28 and 4.20, on the same carbon at δ_C_ 38.6 and showing long range correlations with a pyrone ring (C-1 and C-4) and with an aromatic ring (C-8 and C-12). A second methylene unit was deduced by the presence of two doublets at δ_H_ 2.89 and 2.79, both HSQC correlated with the same carbon at δ_C_ 47.1 and HMBC correlated to C-12, C13, C-15 and the alkyl C-16 position. The last evidence gave indication also for the *n*-propyl chain at C-15 position, confirmed by the long range correlation of protons at C-16 (δ_H_ 1.88) with C-14 and C-16.

**Table 1:** NMR spectroscopic data for compound **13** (400 MHz, in CDCl_3_ and CD_3_OD). δ in ppm, *J* in Hz.

Position	δ_H_ (CD_3_OD)	δ_H_ (CDCl_3_)	δ_C_, (CDCl_3_)	HMBC ^a^
1	–	–	165.4	
2	–	5.33, s	89.9	3, 4, 5
3			171.3	
4	5.65, s	5.75, s	101.2	
5			166.9	
6	4.23, dd(16.2)	4.20, d(16.4)	38.6	1, 4, 8, 12
	4.32, dd(16.2)	4.28, d(16.4)		
7			133.5	
8	6.99, d (8.6)	6.99, s	125.2	
9	7.48, t (8.1)	7.39, t (8.2)	135.4	7, 11
10	6.93, d (7.2)	6.90, d(8.2)	118.4	8, 11, 12
11			158.1	
12			118.5	
13			194.0	
14	–	2.79, d (16.0)	47.1	12,13,15, 16
	–	2.89, d (16.0)		
15			102.3	
16	1.89, m	1.88, m	43.1	14, 15, 17, 18
17	1.53, m	1.57, m	17.1	15, 16, 18
17	1.00, t (7.0)	0.99 (t, 7.0)	14.04	17, 16

^a^ HMBC correlations are from protons(s) in the line with the indicated carbon(s).

If compared to the one recorded in CDCl_3_,^1^H NMR spectrum of **13** in CD_3_OD showed a fast disappearance of some signals, in detail the singlet assigned at H-2 and the two doublets for the protons at C-14 are lacking (Supplementary Figure S4). Otherwise, the ESI-MS spectrum recorded in positive ion mode detection for the same CD_3_OD solution of **13** showed a signal at *m/z* 358; comparing it with the corresponding signal at *m/z* 353 attributed to [M + Na]^+^ by direct injection of a methanol solution of **13**, it was possible to deduce the incorporation of five deuterium atoms in the molecule (Figure 2). Fragmentation MS/MS experiments on *m/z* 358 gave diagnostic signals: (i) at *m/z* 338 for the loss of a D_2_O molecule (in agreement with the corresponding fragment at *m/z* 335 produced from *m/z* 353, Experimental Section) indicating the H/D exchange commonly occurring for the alcohol group at C-15, and for the protons at C-14 by a favored equilibrium with the enol form stabilized by conjugation with an aromatic ring; (ii) at *m/z* 245 deriving from the loss of a pyrone fragment bearing two deuterium atoms by the breakage in C5-C6 position ((Supplementary Figure S7). These results were confirmed by the data from ESI-MS spectrum in negative ion mode, where the signal at *m/z* 332 attributed to [M − D]^−^ molecular ion for deuterated **13** was observed ((Supplementary Figure S8). This behavior of compound **13** is due to the possibility of hydroxypyrones to exist in two tautomeric forms: 4-hydroxy-α-pyrone (=2*H*-pyran-2-one) and 2-hydroxy-γ-pyrone (=4*H*-pyran-4-one), for which the deuterium exchange was observed [17]. Although compound **13** presented no appreciable optical activity, the weak Cotton effect observed by circular dichroic analysis spoke for the chiral property of the natural molecule.

Compound **13** is structurally similar to the previously reported polyketide SEK34 (**3**, Figure 1) by engineering biosynthesis [11]. NMR data of the two products showed that they differ only in the alkyl chain at the position of C-15, and on this basis, we can suggest the γ-pyrone form for this structure. Compound **13** represents the lacking member in the recently reported series of phaeochromycins A–E (**7**–**11**), which are the first polyketides bearing the *n*-propyl chain [14]. A strictly related compound corresponding to the 4-hydroxy α-pyrone form of **13** was reported with a poor structural elucidation, as a shunt product from the engineered system for the production of analogues of frenolicin B [18], so that **13** is the first example from marine derived actinomycetes.

Compound **7**, structurally characterized by extensive NMR analysis and ESI-mass spectrometry (Experimental Section), was proven to be phaeochromycin B [14]. However, the assignment of a pyrone unit could not be unambiguously established by NMR correlations [14], so that further evidence avails; for this reason the method discussed below is proposed.

Similarly, structural analysis on compound **9** allowed for establishing that it was phaeochromycin C [14], even if the same ambiguity for the tautomeric pyrone assignment persisted.

Compound **11** resulted to be phaeochromycin E, through comparison of acquired NMR data, including HSQC and HMBC correlations, with these ones reported [14]. ESI-MS analysis, both in positive and negative ion mode, supported the molecular composition. 

### 2.2. Hydroxypyrone Tautomerism

The reported γ-pyrone form for mutactin (**1**, Figure 1) was confirmed by X-ray analysis and by ^2^*J*_C,H_ coupling constants observed for C-3 (δ_C_ 170 in acetone-*d*_6_) with both H-2 and H-4, whereas C-1 (δ_C_ 164.4) was coupled only to H-2 [10]. Successively, the same tautomeric form was established for the hydroxypyrone unit in SEK34 (**3**) structure, based on similar ^13^C NMR chemical shifts with mutactin [11]. Further reports of these molecular structures and new hydroxypyrone polyketides as phaeochromycins A–C (**7**–**9**, Figure 1) drew them in the α-pyrone form [14]. Based also on a general interest in the pyranone tautomerism [19], there is the need to look deeper into the structural study for this class of metabolites, with the aim of defining a method able to assign the right α- or γ-pyrone form.

Over the last decades, infrared spectroscopy has experienced a renewed role in the molecular structure elucidation of natural products, due to the availability of simulated spectra by density functional theory (DFT) calculations, used to assign the frequencies of chemical bonds through comparison with experimental data. IR spectra was calculated at a B3LYP/6-31G(*d*,*p*) level of theory for compounds **7**, **9** and **13**, both in 4-hydroxy-α-pyrone and in 2-hydroxy-γ-pyrone forms, and compared to the experimental FT-IR spectra ((Supplementary Figures S12–S17). In Table 2 the C=O values present in the structures of these molecules are reported. 

**Table 2:** DFT calculated stretching frequencies (in cm^−1^) for the carbonyl groups in α-pyrone (**A**) and γ-pyrone (**B**) forms and experimental values for **7**, **9** and **13** and 4-hydroxy-6-methyl-2*H*-pyran-2-one as reference compound. Arbitrary numbering on A and B forms is for convenience.

		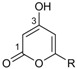 A		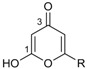 B	
		ν_IR_calcd			
Compound	ν_IR_exp.	C(1)=O	C(13)=O ^a^	C(3)=O	C(13)=O ^a^
7	1637	1780	1639	1686	1642
	1620				
	1578				
	1524				
9	1699	1780	1649	1683	1662
	1645				
	1607				
	1598				
13	1689	1782	1678	1682	1698
	1649				
	1603				
	1593				
R=CH_3_^b^	1738	1782	–	1692	–
	1645				
	1534				

^a^ According to numbering in the structures reported in Figure 1; ^b^ Experimental values from [20].

It is evident that the simulated frequency for carbonyl stretching appears at 1780–1782 cm^−1^ in the α-pyrone form A and at 1682–1686 cm^−1^ in the γ-pyrone form B. The comparison with the experimental FT-IR spectra recorded for **7**, **9** and **13** (showing no frequencies over 1700 cm^−1^) indicates that they may be described in the 2-hydroxy γ-pyrone form. In order to confirm that the method is plausible, commercial 4-hydroxy-6-methyl-2*H*-pyran-2-one was used. By comparing the calculated C=O frequencies for its two tautomeric forms with the experimental value (in agreement with the one reported [20]), it resulted in line with the known α-pyrone structure for this compound.

### 2.3. Antibacterial Evaluation

Promising activity against Gram positive *S. aureus* and methicillin resistant *S. aureus* (MRSA) and Gram negative *E. coli* and *P. aeruginosa* were observed by antimicrobial assays on the crude extract of the actinomycete WR1L1S8 strain, accounting for the evaluation on the pure isolated compounds **9**, **11** and **13**. They were tested preliminary on five bacterial test organisms, using the agar diffusion method. The compounds displayed similar activities toward the tested bacteria, with weak inhibition effects ((Supplementary Table S1), and a bacteriostatic activity against MRSA by compound **13**. In a further investigation in order to define MIC values against MRSA, *E. coli* and *P. aeruginosa*, interestingly **13** showed selective antimicrobial activities (MIC = 2 μg/mL = 6.0 μM, Table 3) against MRSA, a bacterium responsible for human infections which is difficult to treat, showing resistance to penicillins and cephalosporins and mainly found among people with a greater risk of infection. Current findings are in line with the activities against *S. aureus* and MRSA bacteria, observed for the 4-hydroxy α-pyronepolyene, gombapyrone E [9]. It is noteworthy that compound **13** showed selective antibacterial activity against MRSA, but no growth inhibition against *S. aureus* ATCC 25923. This can be suggest that compound **13** could probably inhibit the production of Penicillin Binding Protein (PBP2a), encoded by the gene selectively involved in oxacillin or methicillin resistance *S. aureus* [21]. A similar behavior was reported for epicatechin gallate [22].

**Table 3:** Minimum Inhibitory Concentration (MIC) values of compounds **9**, **11** and **13** on some pathogenic strains.

MIC (μg/mL)			
Compounds	*E. coli* ATCC 25922	MRSA ATCC 43300	*P. aeruginosa* ATCC 27853
**9**	–	>32	>32
**11**	>32	>32	>32
**13**	16	2	>32
Vancomycin	–	<2	–
Gentamicin	<2	<2	–

## 3. Experimental Section

### 3.1. General Experimental Procedures

All evaporations were carried out at room temperature under reduced pressure. Solvents (Merck, Milan, Italy) were used without purification. Flash chromatography (FC) was carried out on *Merck* Si-60 (15–25 μm) using hexane/ethyl acetate or dichloromethane/methanol gradient elution as mobile phase. TLC was performed on *Merck* silica gel 60 F*254* and spots could be seen using UV light (λ 254 and 365 nm) and spraying with anisaldehyde/sulfuric acid reagent followed by heating. Preparative thin layer chromatography was carried out on 20 × 20 cm *Merck* Kiesel gel 60 F_254_ 0.5 mm plates. Analytical HPLC-DAD-ELSD experiments were performed using a reversed phase C18 column (Hewlett Packard Hypersil BDS-C18, 250 × 4.00 mm). The mobile phase was applied as the linear gradient. Chromatographic parameters are the following: Detection—DAD 210 nm; mobile phase A acetonitrile, B water; 0–10 min 30% A–70% B, 10–30 min 100% A, flow rate 1 mL/min. Polarimetric data were obtained using a Bellingham & Stanley Limited ADP 440 apparatus, reporting [α]_D_ in dm^−1^·deg mL g^−1^. Cotton effects were deduced by circular dichroic (CD) spectra, recorded by a Jasco J-710 spectropolarimeter. Infra-red spectra were recorded by using a FT-IR Tensor 27 Bruker spectrometer (Attenuated Transmitter Reflection, ATR configuration) at 1 cm^−1^ resolution in the absorption region 4000–1000 cm^–1^. A thin solid layer is obtained by the evaporation of a methanol solution of the sample. The instrument was purged with a constant dry air flux and clean ATR crystal as background was used. Spectra processing was made using Opus software package. NMR spectra were recorded with an Avance 400 Bruker spectrometer by using a 5 mm BBI probe; ^1^H at 400 MHz and ^13^C at100 MHz in CDCl_3_ (by previous treatment on basic alumina to avoid acidic traces), CD_3_OD or acetone-*d*_6_, δ values in ppm, relative to the solvent residual signals δ_H_ = 7.25 ppm for CDCl_3_, δ_H_ = 3.31 ppm for CD_3_OD, δ_H_ = 2.06 ppm for acetone-*d*_6_ where the solvent residual signals are relative to SiMe_4_ (=0 ppm); *J* values in Hz. Structural assignments are from Heteronuclear Single Quantum Correlation (HSQC) and Heteronuclear Multiple Bond Correlation (HMBC) experiments. NMR assignments were carried out following an arbitrary numbering (adopted for convenience), as reported in Figure 1 and Figure 2. Electron-impact (EI) mass spectra (*m/z*; rel.%) and HR-EI data were taken with a Kratos-MS80 mass spectrometer with home-made computerized acquisition software. ESI-MS data and tandem fragmentation spectra (MS/MS) were recorded by using a Bruker Esquire LC ion trap mass spectrometer, equipped with an ESI ion source in positive or negative ion mode as specified, through injection of the sample into the source from a methanol solution. MS conditions: source temp. 300 °C, nebulizing gas N_2_, 4 L/min, cone voltage 32 V, scan range *m*/*z* = 100–800. Fragmentation experiments were carried out by using helium to collisionally activate the selected primary ions.

### 3.2. Isolation and Identification of Streptomyces Strain WR1L1S8

Brown algae of the genus *Fucus* were collected in Bejaia coastline, Algeria (36°39′4.25″N; 5°25′3.88″E). Liquid tissue portions were prepared and used for the isolation of actinomycetes. Samples were spread on selective agar plates in triplicate Petri plates, within 24 h after collection, and were incubated at 28 °C for three weeks. Bacteria colonies were selected with sterile needles and purified by streaking them out onto Starch Casein Agar plates (SCA). After another seven days of growth, the only colony (strain WR1L1S8) that grew well and presented a relatively interesting antibacterial activity was transferred either to the same medium agar slants for short term storage at 4 °C and in 20% glycerol at −80 °C for long-term storage. The actinomycete strain was identified according to morphological physiological and chemotaxonomic studies. The molecular identification was determined by the partial 16S rRNA gene by direct sequencing of amplified 16S rDNA (DSMZ, Braunschweig, Germany).

### 3.3. Isolation and Structural Characterization of Compounds ***7**, **9**, **11*** and ***13***

Cultivation was carried out on 100 SCA plates using ASF process rather than liquid media (because of the apparently more abundant production of metabolites on agar media) and incubated at 28 °C for seven days. Mycelial mass together with the agar were cut into small pieces then mixed; they were thereafter sonicated in a sonication bath for 1 h and macerated overnight with 200 mL of ethyl acetate. The resulting solution was filtered using Whatman filter paper and the same maceration step with ethyl acetate was repeated. The resulting ethyl acetate broth extracts were combined and subsequently dried in a vacuum evaporator, to give the crude extract (500 mg), which was suspended in methanol (3 mL). The ethyl acetate extract was subjected to silica gel column chromatography using a gradient of hexane/ethyl acetate followed by dichloromethane/methanol, to afford 31 fractions. 

Fraction 23 (36.5 mg), eluted with dichloromethane/methanol 8:2 (v/v) was purified further by preparative TLC using hexane/isopropanol 7:3 v/v as mobile phase to yield the violet compound **13** (13.6 mg; 2.72% from crude extract; *R*_f_ = 0.53) and **9** (5.6 mg; 1.12% from crude extract; *R*_f_ = 0.59). Their purity was confirmed by analytical RP-HPLC DAD at 245 nm using a gradient of acetonitrile/water 3:7 (*t*_R_ = 10.6 and 15.5 min, respectively).

Combined fractions 24–25 (47.8 mg, eluted with dichloromethane/methanol 7:3 and 6:4, respectively), were further purified by preparative TLC on silica gel (ethyl acetate/isopropanol 7:3) to give pure compounds **11** (2.6 mg; 0.52% from crude extract ; *R*_f_ = 0.52) and **7** (3.7 mg; 0.74% from crude extract; *R*_f_ = 0.38). Their purity was confirmed by RP-HPLC DAD analysis at 245 nm (*t*_R_ = 2.71 and 3.72 min, respectively).

Compound **7**: Colorless amorphous glass. IR: 2962, 2959, 2900, 1637, 1620, 1578, 1524, 1468, 1408, 1373, 1358, 1242, 1157, 822 cm^−1^. NMR data (in acetone-*d*_6_): in agreement with the reported data [14]. ESI (+)-MS: *m/z* 353 [M + Na]^+^; MS/MS (353): *m/z* 335, 311, 241 and 222; ESI (−)-MS: *m/z* 329 [M − H]^−^, 659 [2 M − H]^−^; MS/MS (329): *m/z* 285, 267, 257; MS/MS (659): *m/z* 329.

Compound **9**: Pale brown amorphous glass. IR: 2966, 2933, 2899, 1699, 1645, 1607, 1598, 1477, 1392, 1256, 1174, 968, 852 cm^−1^. NMR (in acetone-*d_6_*): in agreement with the reported data [14]. ESI (+)-MS: *m/z* 335 [M + Na]^+^, 313 [M + H]^+^; MS/MS (335): *m/z* 317, 293 and 249; MS/MS (313): *m/z* 295, 271, 254, 229, 201; ESI (−)-MS: *m/z* 311 [M − H]^−^; MS/MS (311): 296, 267, 249, 225, 201. 

Compound **11**: Pale brown amorphous glass. NMR (CD_3_OD): in agreement with the reported data [14]. ESI (+)-MS: *m/z* 269 [M + Na]^+^, *m/z* 291[M − H + 2 Na]^+^; ESI (−)-MS: *m/z* 311 [M − H]^−^.

Compound **13**: [=2-hydroxy-5-((6-hydroxy-4-oxo-4*H*-pyran-2-yl) methyl)-2-propylchroman-4one]: pale violet amorphous glass. UV (MeOH) λ_max_(ε) 205(3240), 255 (940) and 285(800) nm; CD (MeOH, *c* = 3.6 × 10^−3^ M): Δε (230): +0.012, Δε (258): −0.012 IR: 2927, 2365, 1689, 1649, 1603, 1593, 1471, 1324, 1267, 1166, 1022, 850 cm^−1^. NMR data, see Table 1. ESI (+)-MS: *m/z* 353 [M + Na]^+^; MS/MS(353): *m/z* 335, 311, 293 and 241; ESI (−)-MS: *m/z* 351 [M + Na − 2 H]^−^, 329 [M − H]^−^, MS/MS (329): *m/z* 311, 285, 267 and 243; EI-MS: *m/z* (%): 330 (M^+^, 0.4), 312 (70), 295 (69), 268 (28), 253 (35), 226 (100); HREIMS *m/z* 312.0999 ± 0.0030 (calcd for C_18_H_16_O_5_, 312.0998); 295.0965 ± 0.0030 (calcd for C_18_H_15_O_4_, 295.0970).

### 3.4. Computational Details

Starting structures were generated by molecular mechanics minimization, using PC Model [23]. Quantum chemical calculations were performed using the Gaussian 03W revision E.01 package program set [24]. The standard basis set of choice was 6-31G (*d*,*p*) for all atoms. The gradient-corrected DFT with the three-parameter hybrid functional (B3) [25] for the exchange part and the Lee-Yang-Parr correlation function [26] were utilized. The simulations were made *in vacuo*. The optimized structural parameters were employed in the vibrational energy calculations at the DFT levels to characterize all stationary points as minima. For each optimized structure, no imaginary wavenumber modes were obtained, proving that a local minimum on the potential energy surface was actually found. The computed wavenumbers were scaled by factor 0.96, which is suggested for B3LYP/6-31G (*d*,*p*) calculations [27].

### 3.5. Antibacterial Assay

The antibacterial activities of **9**, **11** and **13** were evaluated by agar dilution assay against several target organisms including Gram-negative bacteria *Escherichia coli* ATCC 25922 and *Pseudomonas aeruginosa* ATCC 27853, as well as Gram-positive bacteria *Staphylococcus aureus* ATCC 25923, *MRSA* ATCC 43300 and *Bacillus subtilis* ATCC 6633*.* Erythromycin (15 μg/disc), gentamicin (10 μg/disc) and vancomycin (30 μg/disc) were used as positive controls.

The pure compounds were dissolved in DMSO (0.7 mg/mL for **9**, 0.5 mg/mL for **11** and 1 mg/mL for **13**). Briefly, assay plates were prepared by inoculating Mueller-Hinton agar medium with 24 h-old culture containing test organisms (10^7^ CFU/mL). 100 μL of dissolved compounds were added to separate wells (6 mm diameter). The plates were placed at 4 °C for 2 h; zones of inhibition were recorded after 24 h of incubation at 37 °C. No growth inhibition was observed for the controls containing only the respective amount of solvent. Antimicrobial activities were assayed in triplicate.

For the purpose of MIC evaluation, each pure compound was dissolved in DMSO at concentrations mentioned above and diluted further to give required concentrations such as 2, 4, 8, 16 and 32 (μg/mL). An inoculum of 10 μL (10^7^ CFU/mL) from culture of each test human pathogens, *E. coli*, MRSA and *P. aeruginosa*, were inoculated in each concentration plate. The plate’s cultures were incubated for 24 h at 37 °C.

MIC was defined as the lowest concentration of the purified compound showing no visible growth after overnight incubation. Vancomycin and Gentamicin were used as positive control for MRSA and *E. coli*, DMSO was used as negative control. Replicates were maintained for each test bacteria.

## 4. Conclusions

In summary, four polyketides including the major one with selective antibacterial activity against MRSA strain were isolated from the algal-derived endophytic actinomycete *Streptomyces sundarbansensis*, which represents the lacking member in the recently reported series of phaeochromycins A–E. We also proposed a method based on the comparison of experimental IR spectra with the DFT ones calculated in order to establish the 4-hydroxy α-pyrone or 2-hydroxy γ-pyrone tautomeric forms for these metabolites. The results indicated a γ-pyrone structure for these compounds, in analogy to the related polyketides mutactin and SEK34.

## Supplementary Files

Supplementary File 1 PDF-Document (PDF, 1078 KB)

## References

[1] Jensen P.R., Mincer T.J., Williams P.G., Fenical W. (2005). Marine actinomycete diversity and natural product discovery. Antonie van Leeuwenhoek.

[2] Lam K.S. (2006). Discovery of novel metabolites from marine actinomycetes. Curr. Opin. Microbiol..

[3] Berdy J. (2005). Bioactive microbial metabolites. J. Antibiot..

[4] Pimentel-Elardo S.M., Kozytska S., Bugni T.S., Ireland C.M., Moll H., Hentschel U. (2010). Anti-Parasitic compounds from *Streptomyces* sp. strains isolated from Mediterranean sponges. Mar. Drugs.

[5] Bredholt H., Fjærvik E., Johnsen G., Zotchev S.B. (2008). Actinomycetes from sediments in the Trondheim Fjord, Norway: Diversity and biological activity. Mar. Drugs.

[6] Stach J.E.M., Maldonado L.A., Ward A.C., Goodfellow M., Bull A.T. (2003). New primers for the class *Actinobacteria*: Application to marine and terrestrial environments. Environ. Microbiol..

[7] De Carvalho C.C.C.R., Fernandes P. (2010). Production of metabolites as bacterial responses to the marine environment. Mar. Drugs.

[8] Jensen P.R., Fenical W. (1996). Marine bacterial diversity as a resource for novel microbial products. J. Ind. Microb. Biotechnol..

[9] Park H.B., Yang H.O., Lee K.R., Kwon H.C. (2011). Gombapyrones E and F, new α-pyronepolyenes produced by *Streptomyces* sp*.* KMC-002. Molecules.

[10] Zhang H.I., He X.G., Adefarati A., Galluci J., Cole S.P., Beale J.M., Keller P.J., Chang C.J., Floss H.G. (1990). Mutactin, a novel polyketide from *Streptomyces coelicoor*. Structure and biosynthetic relationship to Acinorhodin. J. Org. Chem..

[11] McDaniel R., Ebert-Khosla S., Hopwood D.A., Khosla C. (1994). Engineered biosynthesis of novel polyketides: ActVII and actIV genes encode aromatase and cyclase enzymes, respectively. J. Am. Chem. Soc..

[12] McDaniel R., Ebert-Khosla S., Fu H., Hopwood D.A., Khosla C. (1994). Engineered biosynthesis of novel polyketides: Influence of a downstream enzyme on the catalytic specificity of a minimal aromatic polyketidesynthase. Proc. Natl. Acad. Sci. USA.

[13] Xiang L., Kalaitziz J.A., Nilsen G., Chen L., Moore B.S. (2002). Mutational analysis of the enterocinfavorskii biosynthetic rearrangement. Org. Lett..

[14] Graziani E.I., Ritacco F.V., Bernan V.S., Telliez J.B. (2005). Phaeochromycins A–E, anti-inflammatory polyketides isolated from the soil actinomycete *Streptomyces phaeochromogenes* LL-P018. J. Nat. Prod..

[15] Kalaitziz J.A., Moore B.S. (2004). Heterologous biosynthesis of truncated hexaketides derived from the actinorhodin polyketide synthase. J. Nat. Prod..

[16] Arumugam M., Mitra A., Pramanik A., Saha M., Gachhui R., Mukherjee J. (2011). *Streptomyces sundarbansensis* sp. nov., an actinomycete that produces 2-allyloxyphenol. Int. J. Syst. Evol. Microbiol..

[17] Lord R.C., Phillips W.D. (1952). Exchange reactions of γ-pyrone and synthesis of deuterated pyrones. J. Am. Chem. Soc..

[18] Fitzgerald J.T., Ridley C.P., Khosla C. (2011). Engineered biosynthesis of the antiparasitic agent frenolicin band rationally designed analogs in a heterologous host. J. Antibiot..

[19] Goel A., Ram V.J. (2009). Natural and synthetic 2*H*-pyran-2-ones and their versatility in organic synthesis. Tetrahedron.

[20] Seixas de Melo J., Quinteiro G., Pina J., Breda S., Fausto R. (2001). Spectroscopic characterization of α- and γ-pyrones and their substituted 4-hydrxy and 4-methoxy derivative: An integrated infrared, photophysical and theoretical study. J. Mol. Struct..

[21] Tenover F.C. (2006). Mechanisms of antimicrobial resistance in bacteria. Am. J. Med..

[22] Gibbons S. (2004). Anti-Staphylococcal plant natural products. Nat. Prod. Rep..

[23] Gilbert K.E., Midland M.M. (1999). PCMODEL for Windows, version 7.00.

[24] Frisch M.J., Trucks G.W., Schlegel H.B., Scuseria G.E., Robb M.A., Cheeseman J.R., Montgomery J.A., Vreven T., Kudin K.N., Burant J.C. (2004). Gaussian Revision E. 01.

[25] Becke A.D. (1993). Density-functional thermochemistry. III. The role of exact exchange. J. Chem. Phys..

[26] Lee C., Yang W., Parr R.G. (1988). Development of the Colle-Salvetti correlation-energy formula into a functional of the electron density. Phys. Rev. B.

[27] Merrick J.P., Moran D., Radom L. (2007). An evaluation of harmonic vibrational frequency scale factors. J. Phys. Chem. A.

