# Supplementary material for: Antibacterial Polyketides from the Marine Alga-Derived Endophitic Streptomyces sundarbansensis: A Study on Hydroxypyrone Tautomerism
Source: Mar Drugs. 2013 Jan 10;11(1):124–35. doi: 10.3390/md11010124 (PMC3564162; doi:10.3390/md11010124)
Supplement: Supplementary File 1 — PDF-Document (PDF, 1078 KB) [file marinedrugs-11-00124-s001.pdf]

Supplementary Information

Figure S1. ESI (+) MS (top) and fragmentation MS/MS spectraon *m/z* 353 $[M + Na]^+$ (bottom) of compound **13**. 2
Figure S2. ESI (−) MS (top) and MS/MS spectraon *m/z* 329 $[M - H]^-$ (bottom) of compound **13**. 3
Figure S3. ^{1}H-NMR spectrum of **13** in $CDCl_3$. 4
Figure S4. ^{1}H-NMR spectrum of **13** in CD_3OD. 5
Figure S5. HSQC spectrum of **13** in CD_3OD. 6
Figure S6. HMBC spectrum of **13** in CD_3OD. 7
Figure S7. ESI (+) MS and MS/MS spectra on *m/z* 358 $[M + Na]^+$ of deuterated **13** from a CD_3OD solution. 8
Figure S8. ESI (−) MS and MS/MS spectra on *m/z* 332 $[M - H]^-$ of deuterated **13** from a CD_3OD solution. 9
Figure S9. ^{1}H-NMR spectrum of **7** in acetone-d_6. 10
Figure S10. ^{1}H-NMR spectrum of **9** in acetone-d_6. 11
Figure S11. ^{1}H-NMR spectrum of **11** in CD_3OD. 12
Figure S12. DFT calculated IR spectra of 4-hydroxy α-pyrone form (**A**) and 2-hydroxy γ-pyrone form; (**B**) of the compound **7**. 13
Figure S13. Experimental FT-IR spectrum of compound **7**. 14
Figure S14. DFT calculated IR spectra of 4-hydroxyl α-pyrone form (**A**) and 2-hydroxy γ-pyrone form (**B**) of the compound **9**. 15
Figure S15. Experimental FT-IR spectrum of compound **9**. 16
Figure S16. DFT calculated IR spectra of 4-hydroxyl α-pyrone form (**A**) and 2-hydroxy γ-pyrone form (**B**) of the compound **13**. 17
Figure S17. Experimental FT-IR spectrum of compound **13**. 18
Table S1. Antibacterial activities at 100 μL/well of compounds **9**, **11** and **13**. 18

Figure S1. ESI (+) MS (top) and fragmentation MS/MS spectraon *m/z* 353 [M + Na]$^+$ (bottom) of compound **13**.

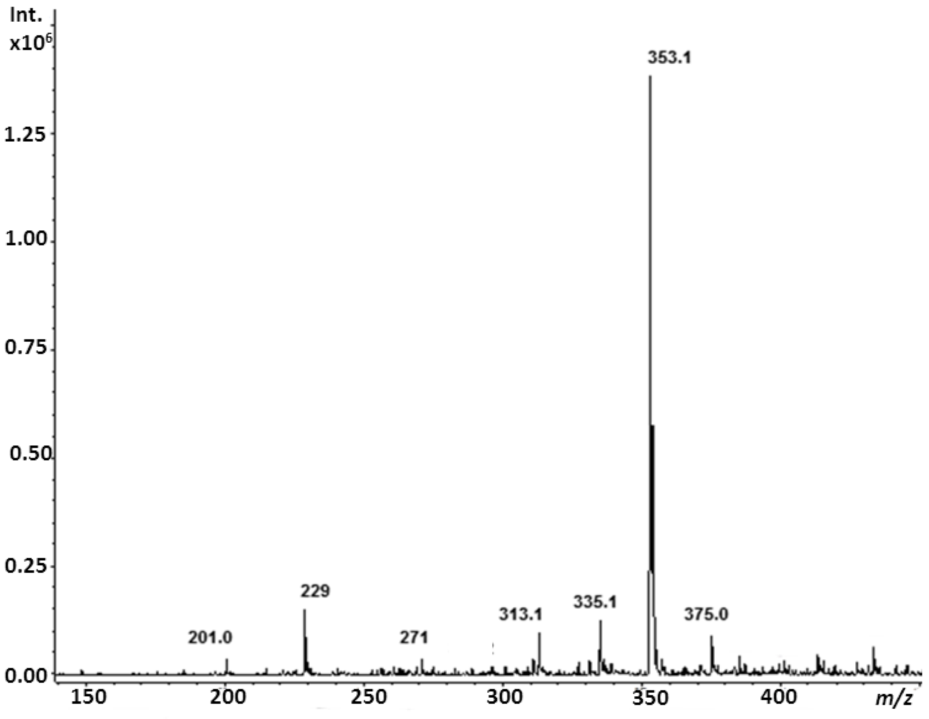

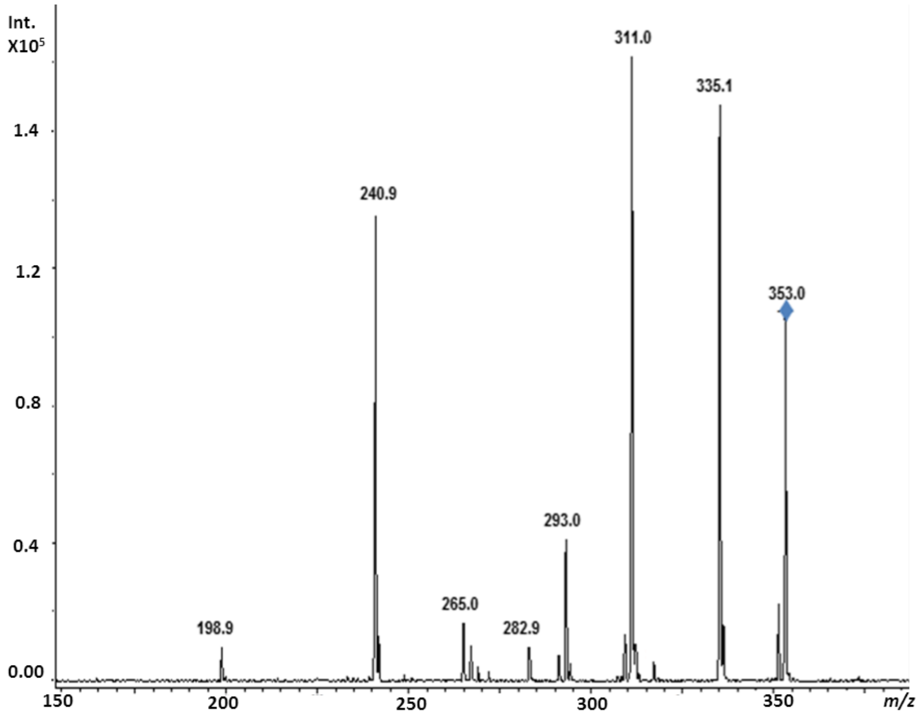

Figure S2. ESI (−) MS (top) and MS/MS spectraon *m/z* 329 [M − H]$^-$ (bottom) of compound **13**.

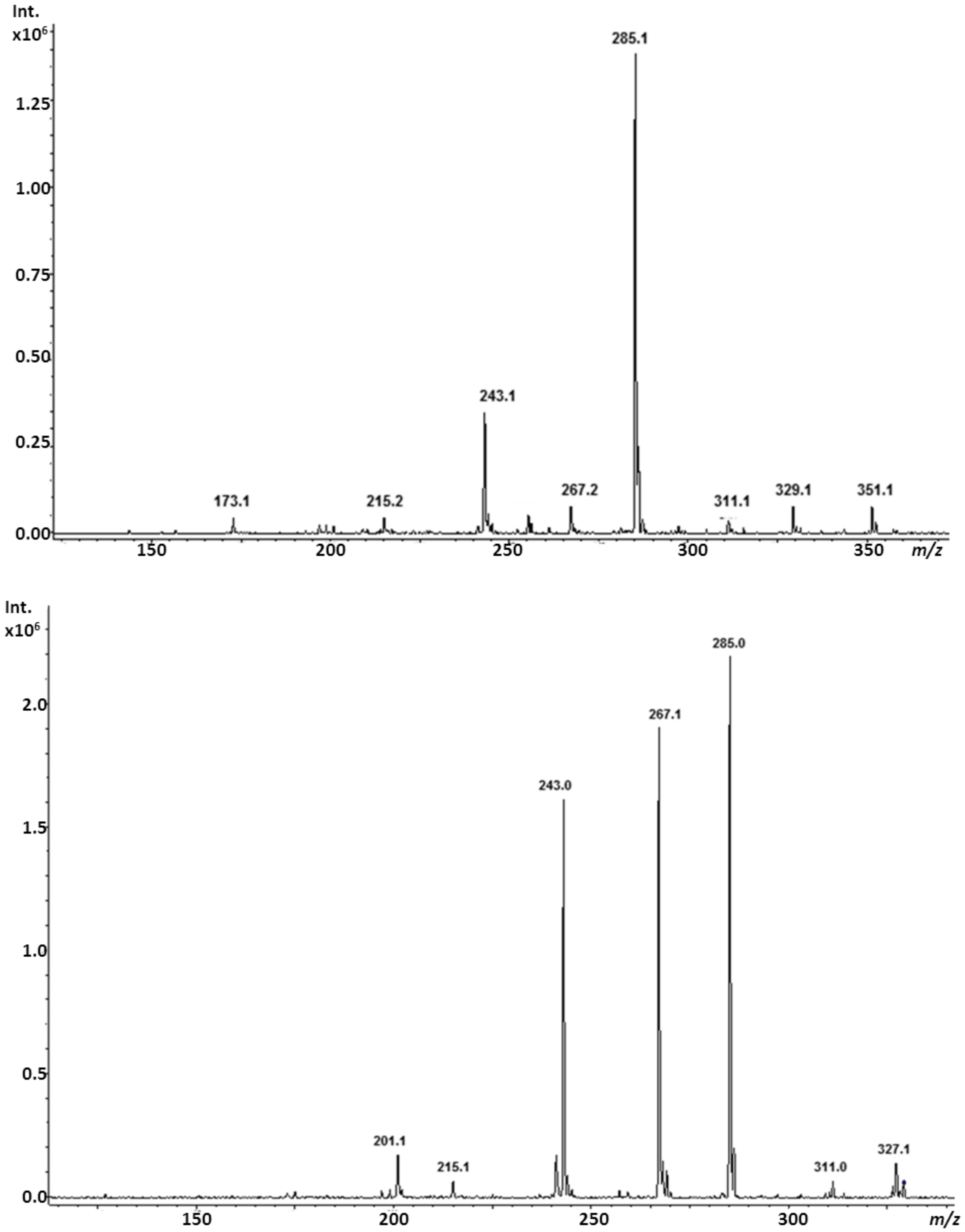

Figure S3. ^{1}H-NMR spectrum of **13** (400 MHz, in $CDCl_3$).

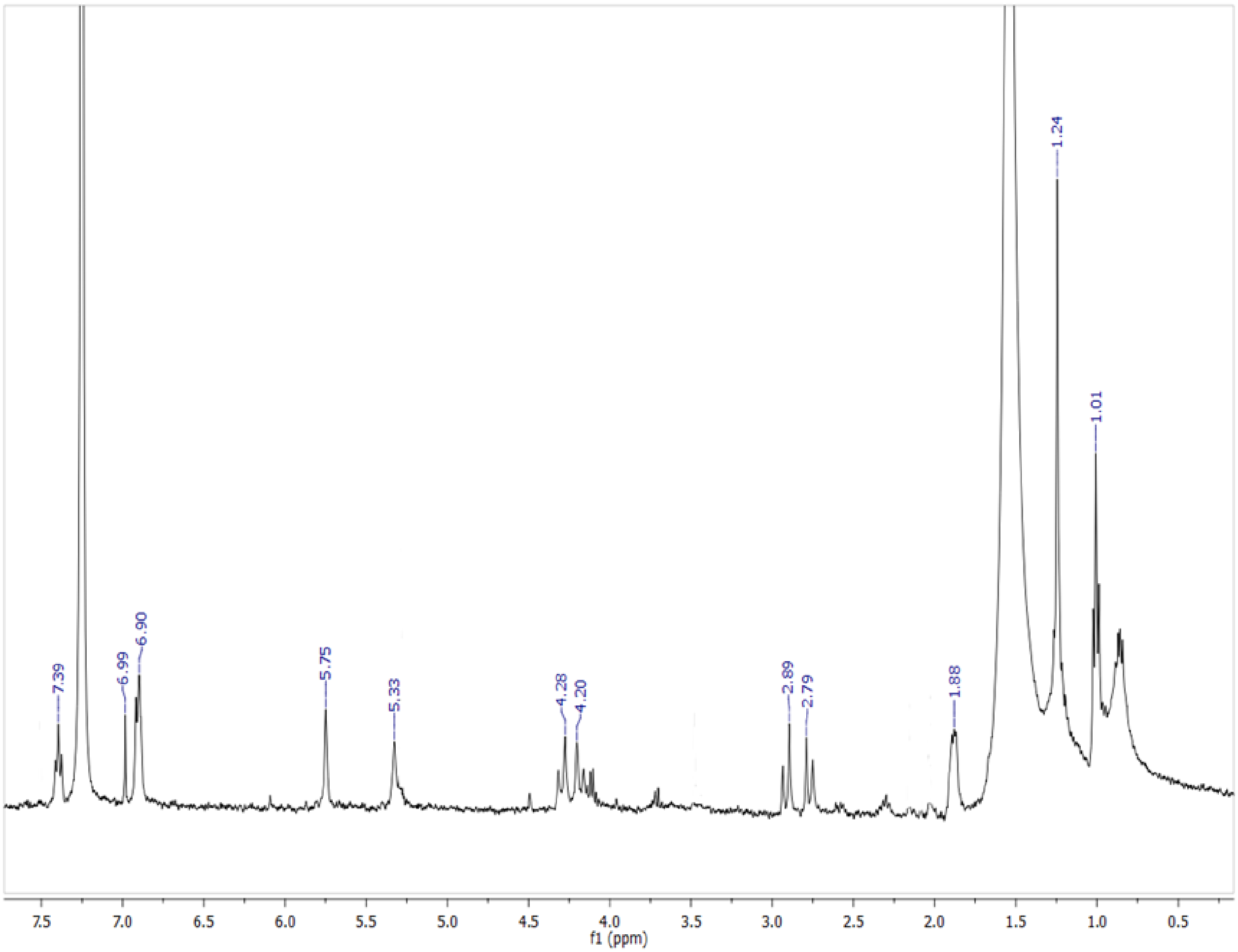

Figure S4. ^{1}H-NMR spectrum of **13** (400 MHz, in CD_3OD).

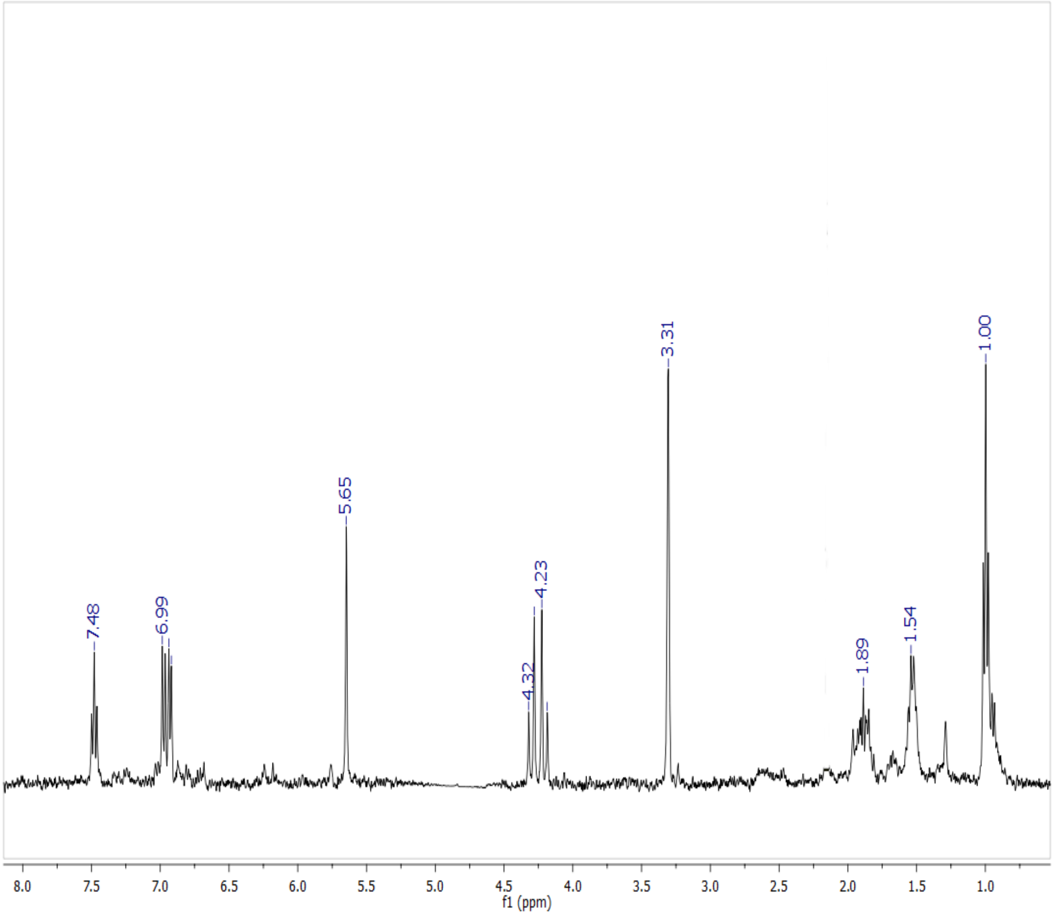

Figure S5. HSQC spectrum of **13** in CD_3OD.

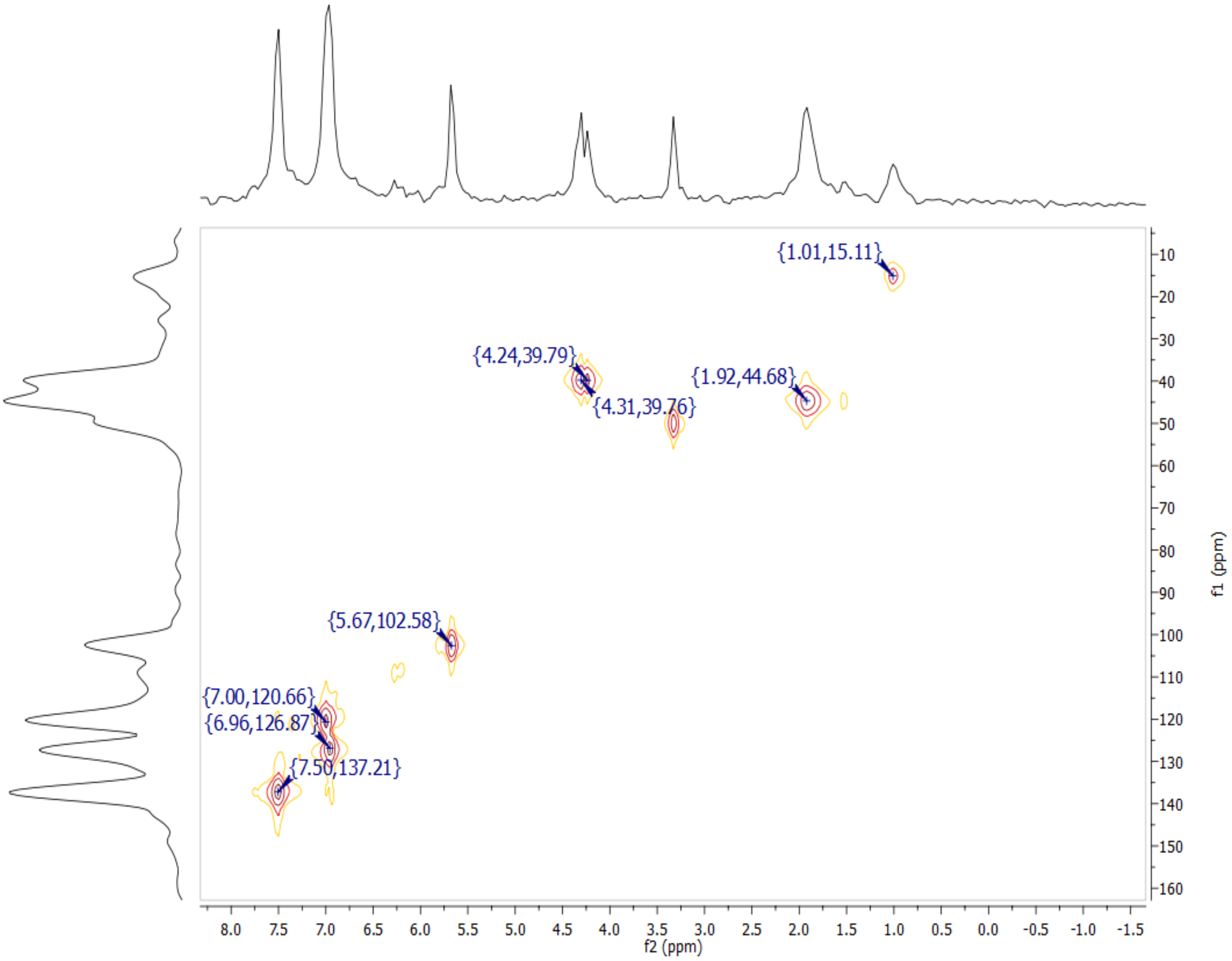

Figure S6. HMBC spectrum of **13** in CD_3OD.

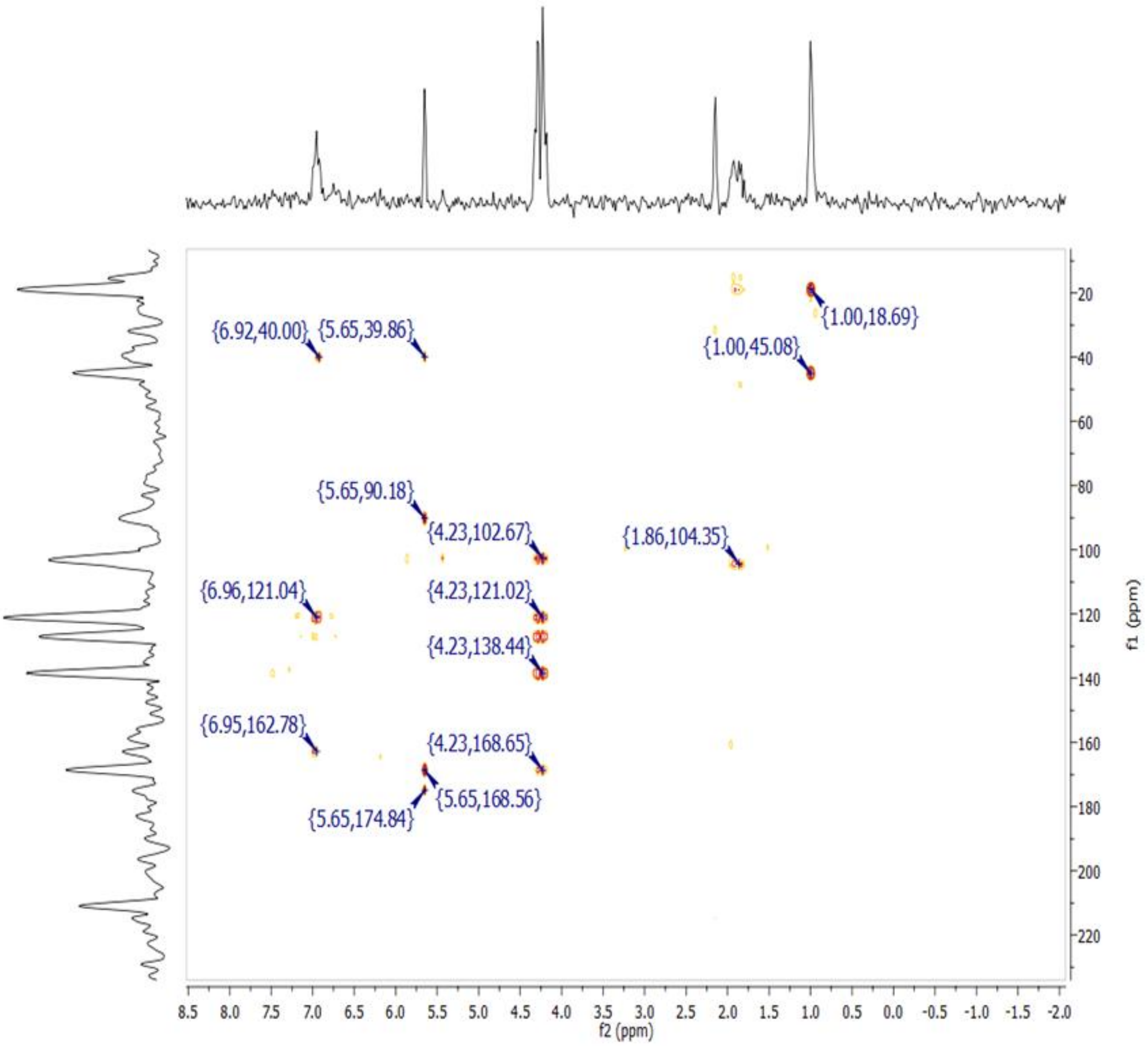

Figure S7. ESI (+) MS and MS/MS spectra on *m/z* 358 $[M + Na]^+$ of deuterated **13** from a CD_3OD solution.

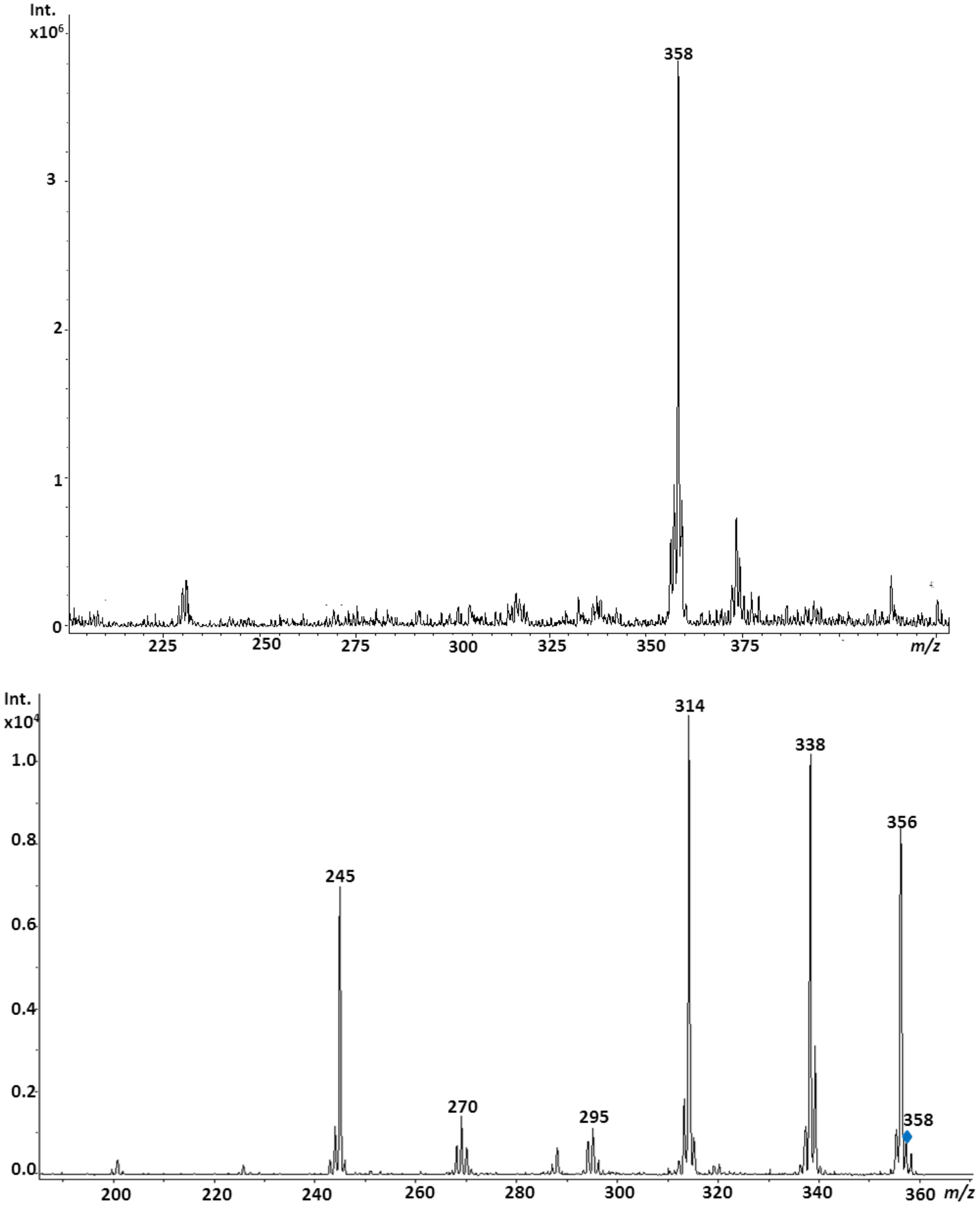

Figure S8. ESI (−) MS and MS/MS spectra on *m/z* 332 $[M-H]^-$ of deuterated **13** from a CD_3OD solution.

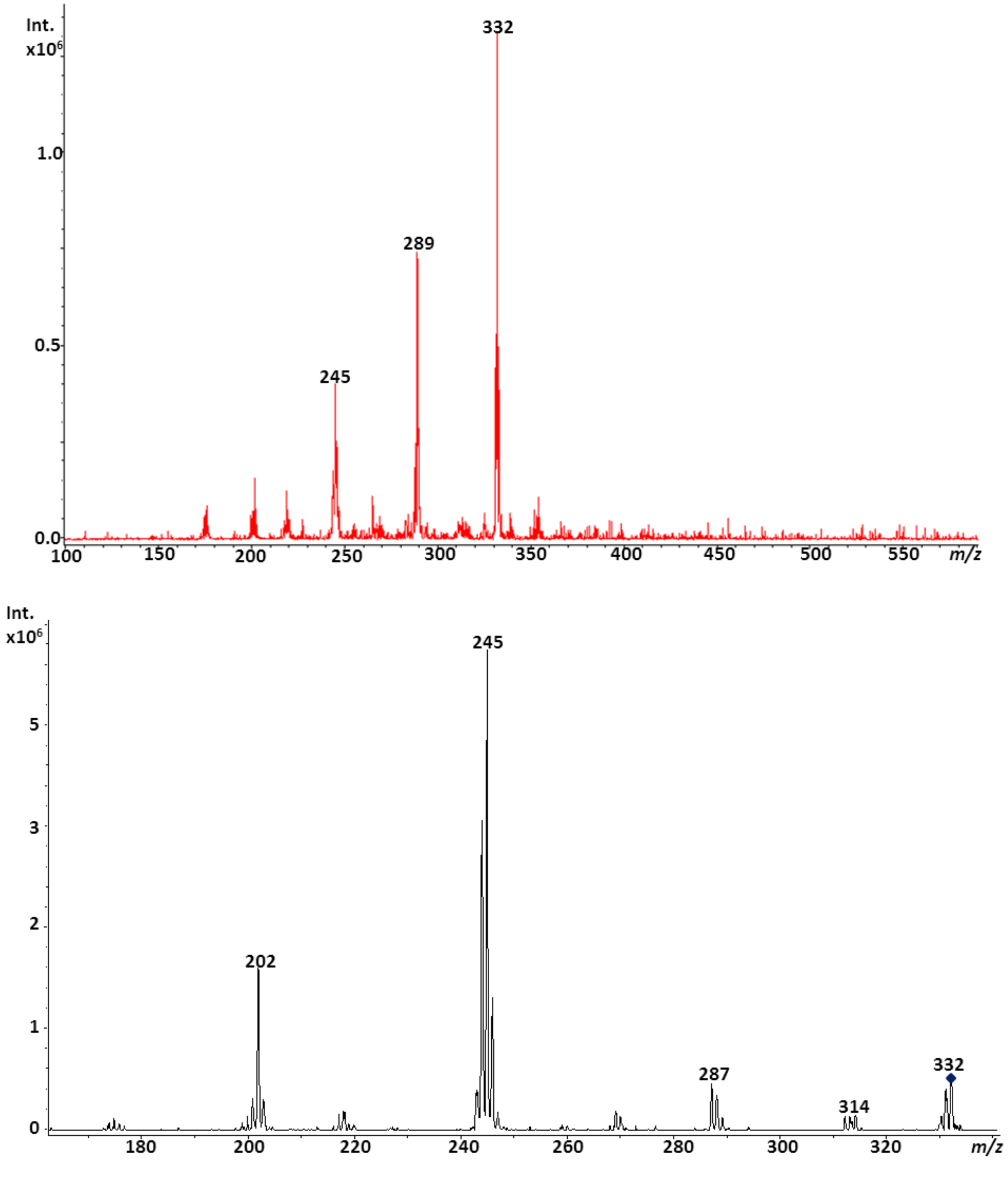

Figure S9. ^{1}H-NMR spectrum of **7** (400 MHz, in acetone-d_6).

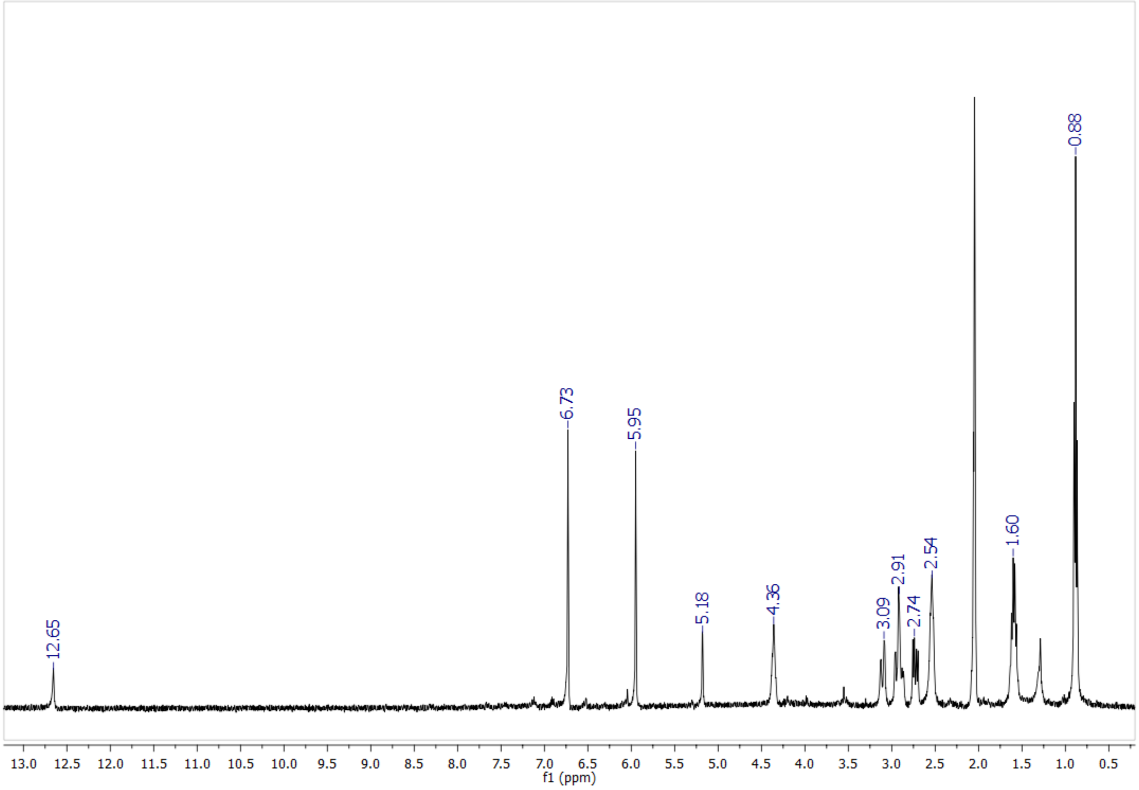

Figure S10. ^{1}H-NMR spectrum of **9** (400 MHz, in acetone-d_6).

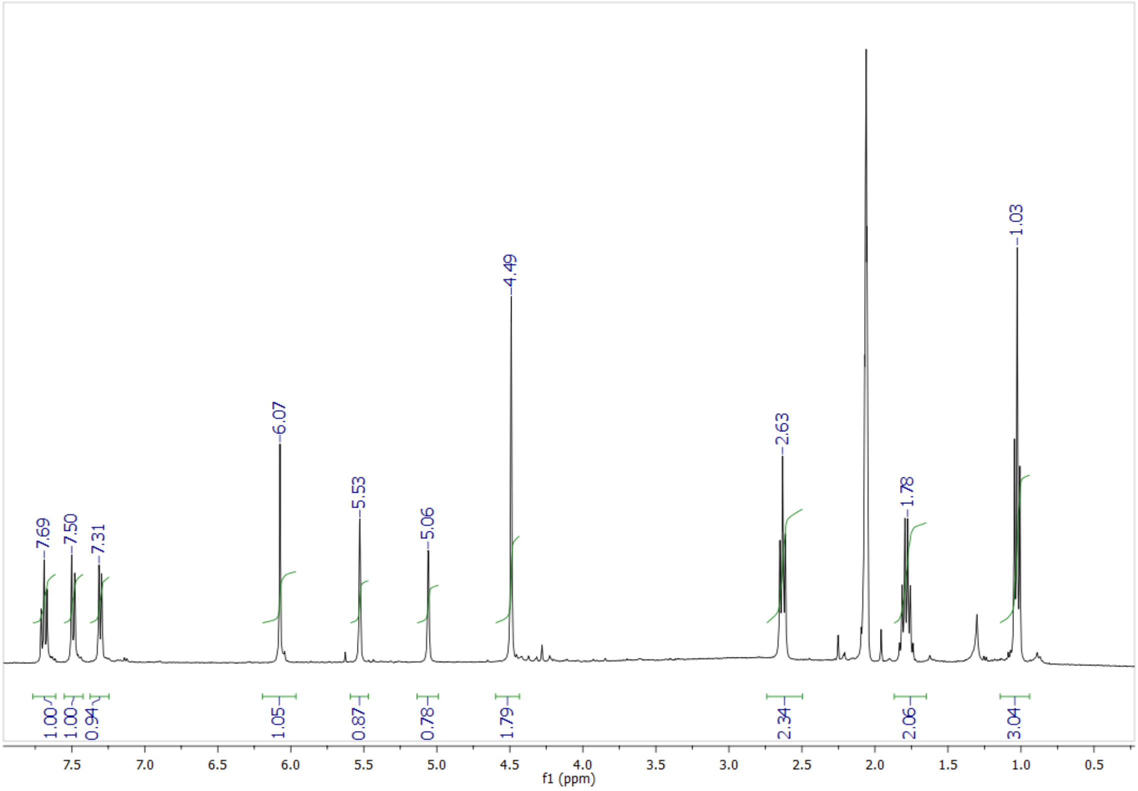

Figure S11. ^{1}H-NMR spectrum of **11** (400 MHz, in CD_3OD).

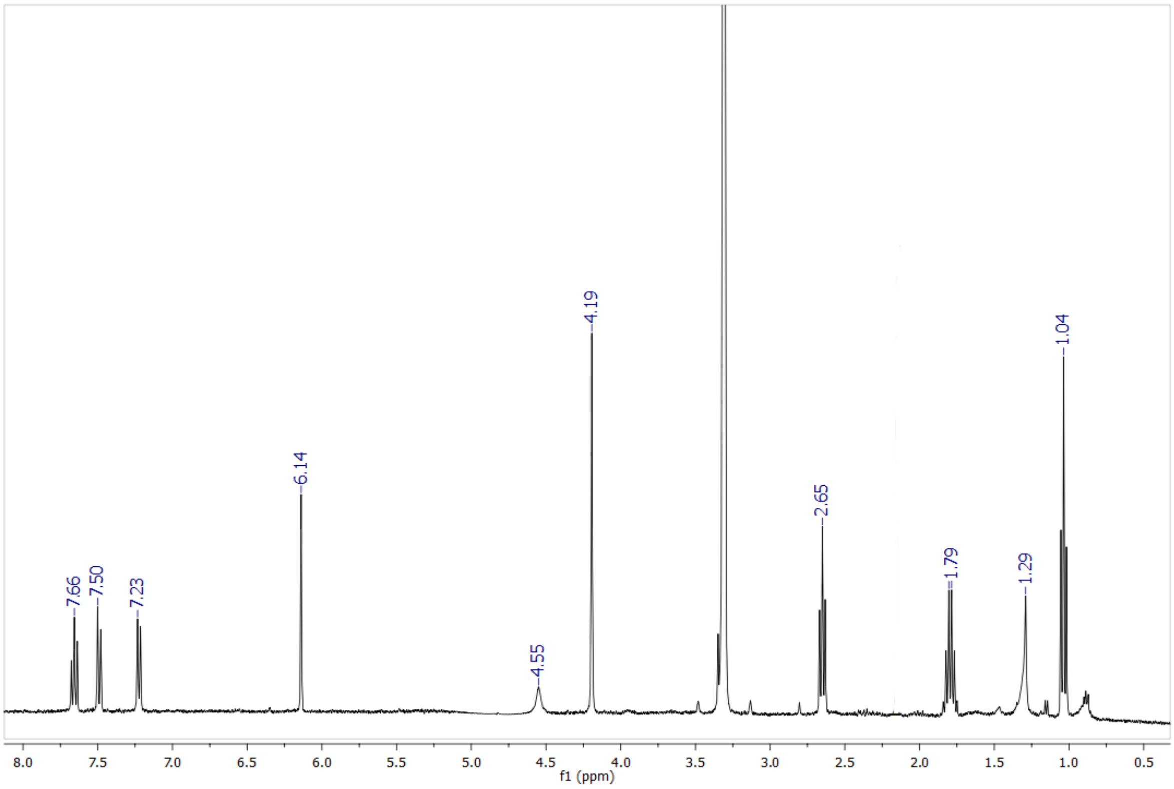

Figure S12. DFTcalculated IR spectra of 4-hydroxy α-pyrone form (**A**) and 2-hydroxy γ-pyroneform (**B**) of the compound **7**.

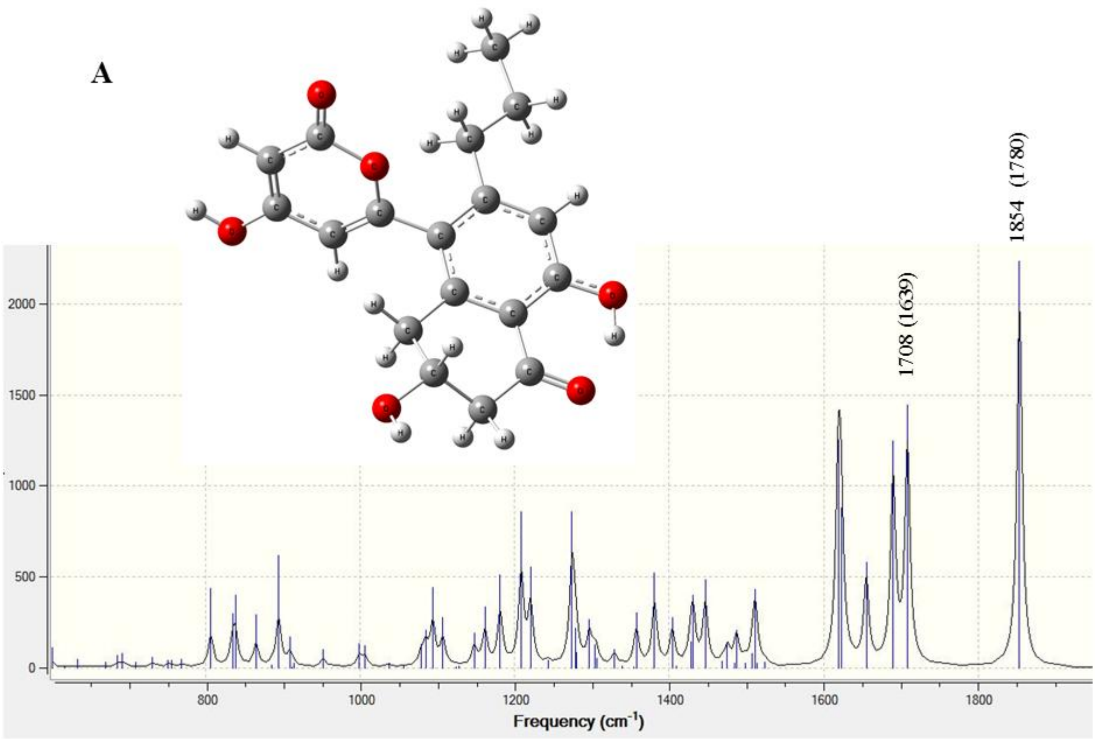

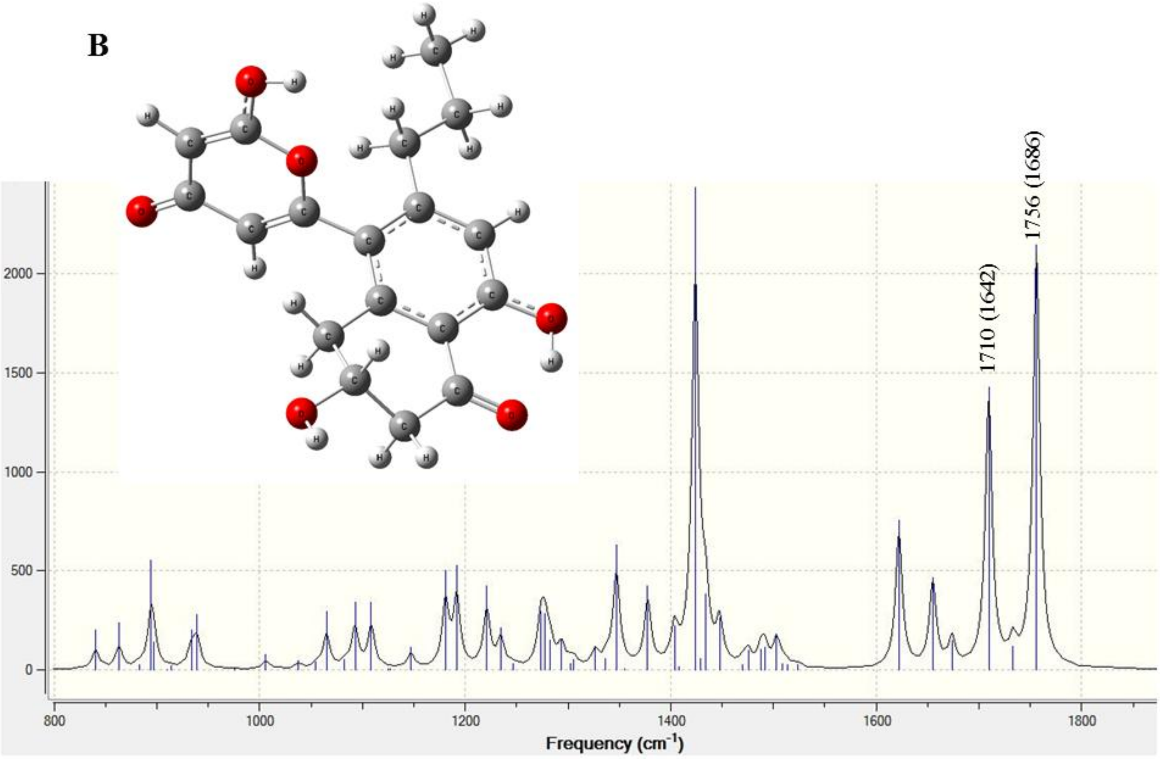

Figure S13. Experimental FT-IR spectrum of compound **7**.

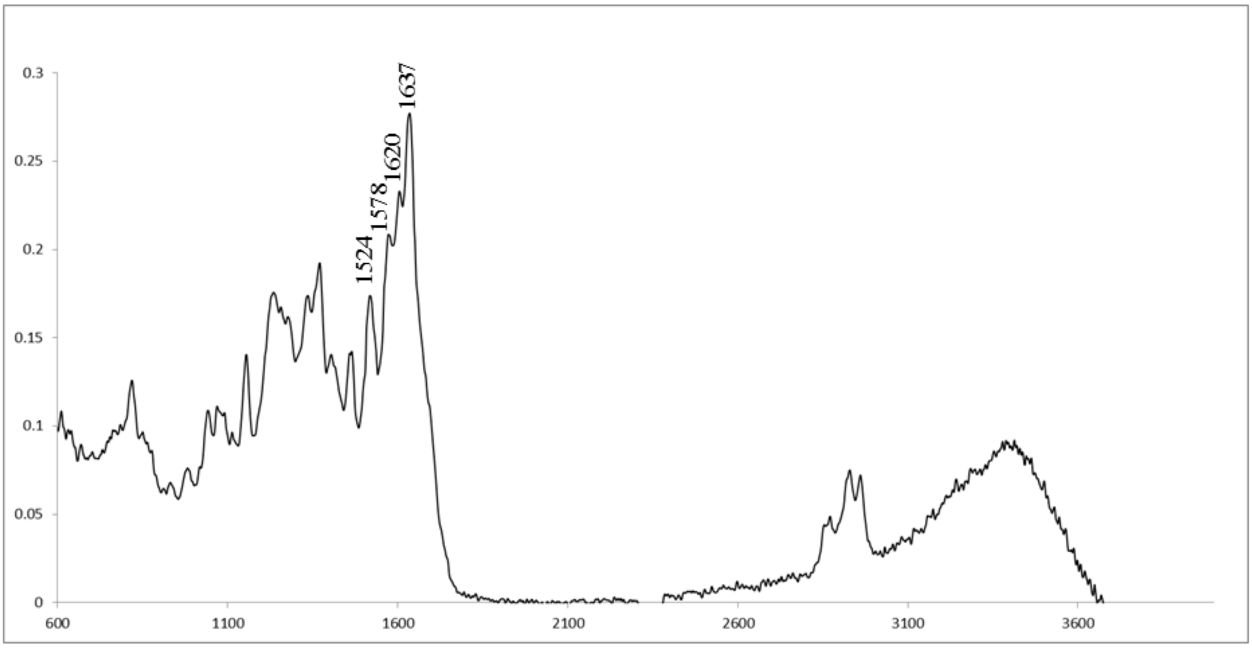

Figure S14. DFT calculated IR spectra of the compound **9** in 4-hydroxy α-pyrone form (**A**) and 2-hydroxy γ-pyroneform (**B**).

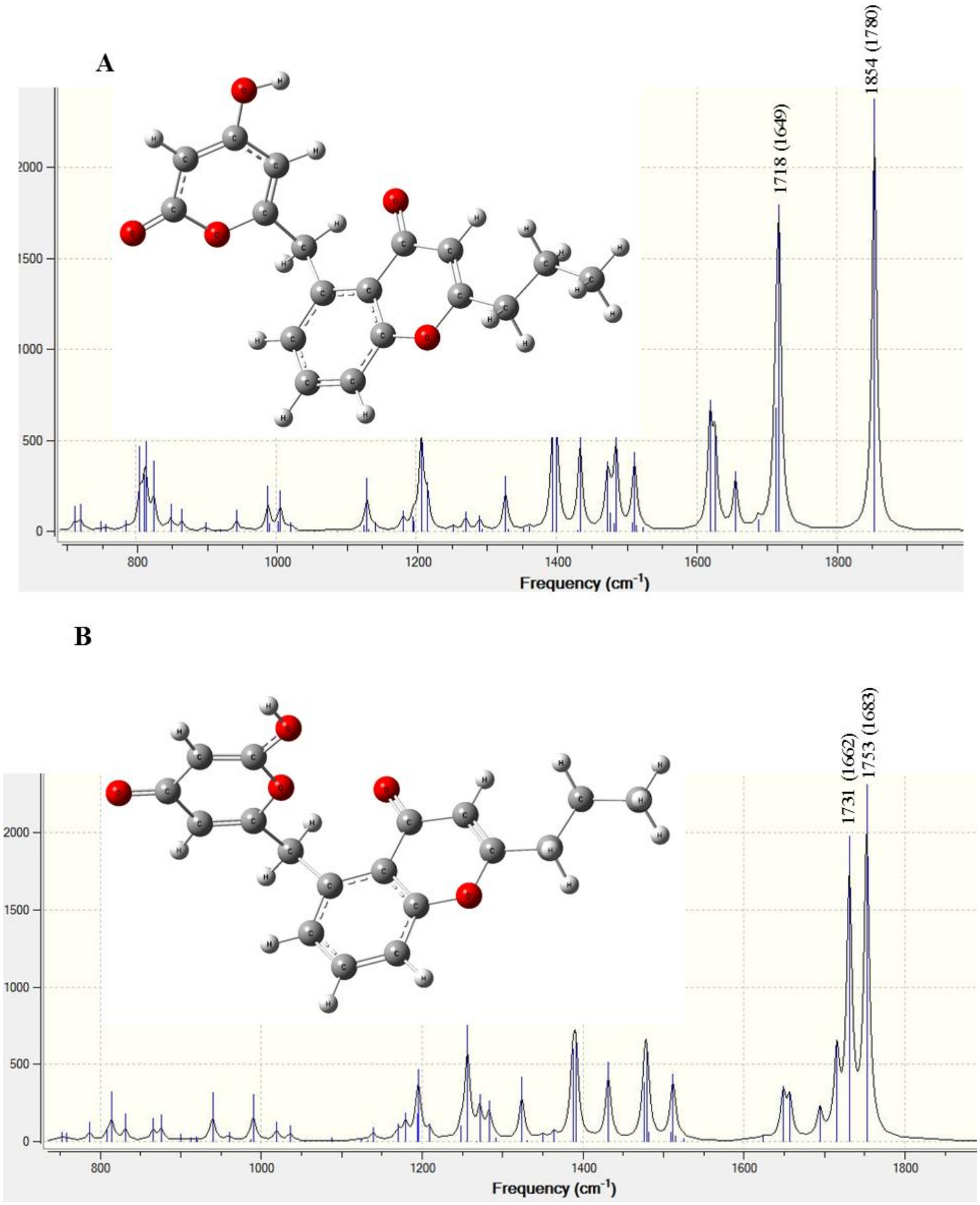

Figure S15. Experimental FT-IR spectrum of compound **9**.

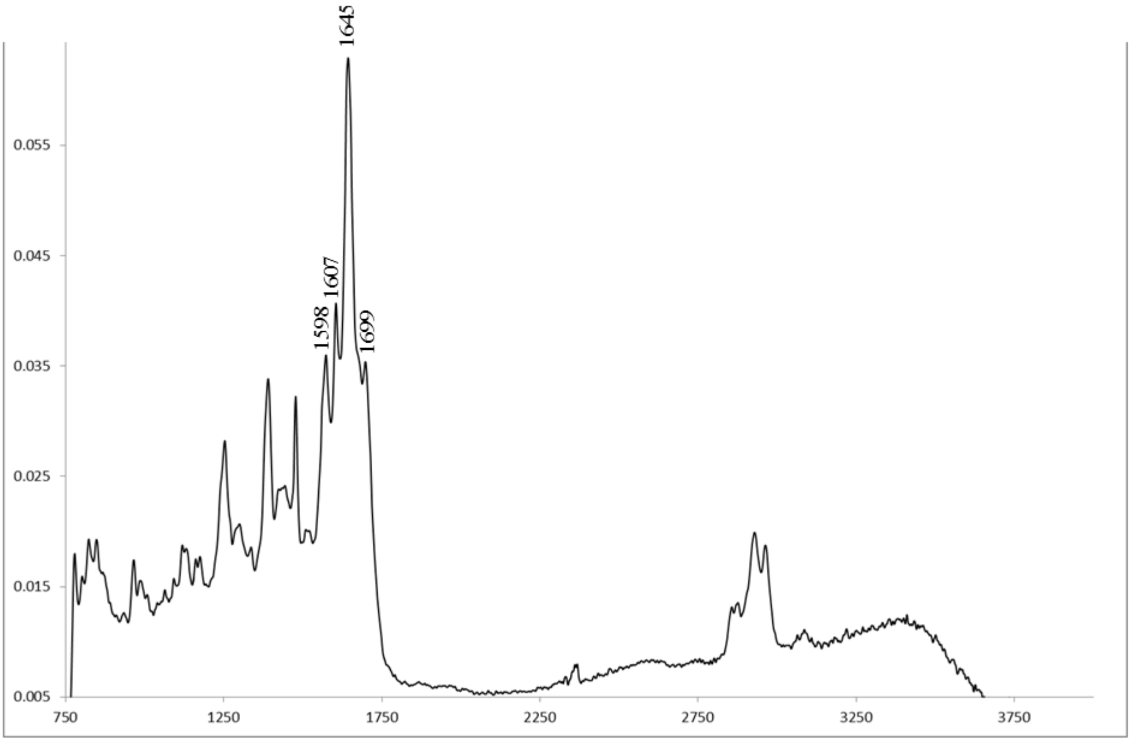

Figure S16. DFT calculated IR spectra of the compound **13** in 4-hydroxy α-pyrone form (**A**) and 2-hydroxy γ-pyrone form (**B**).

A

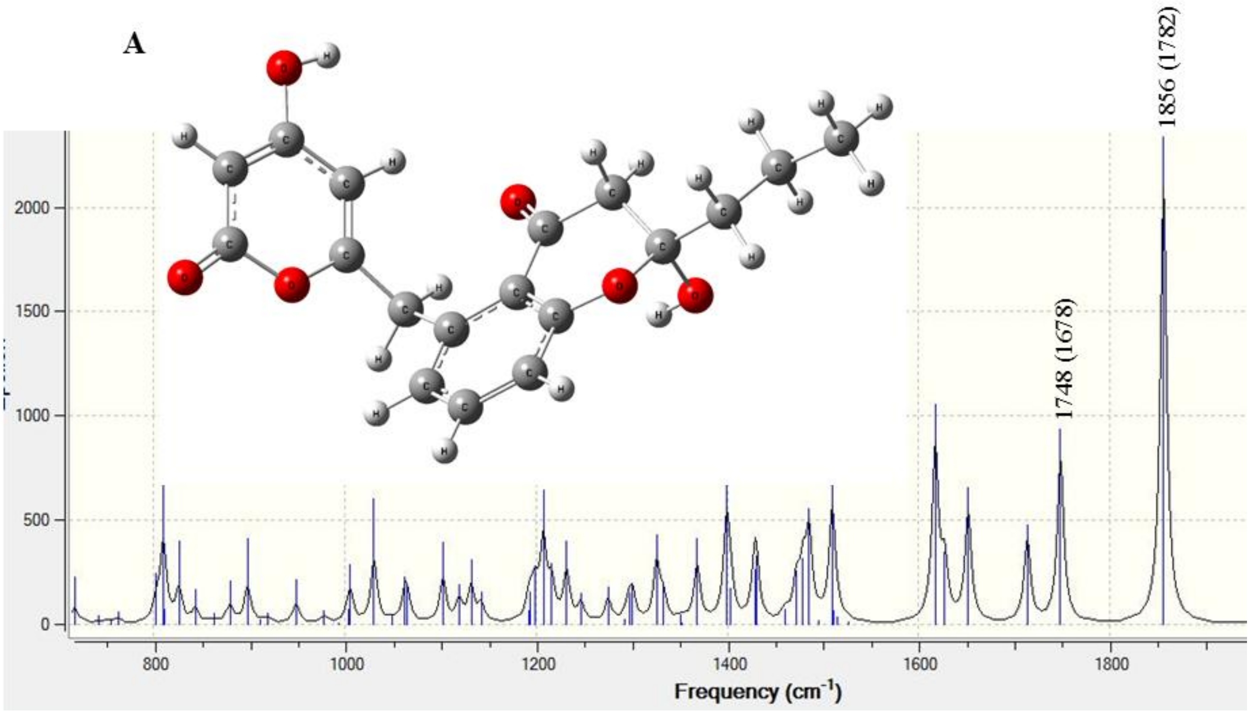

B

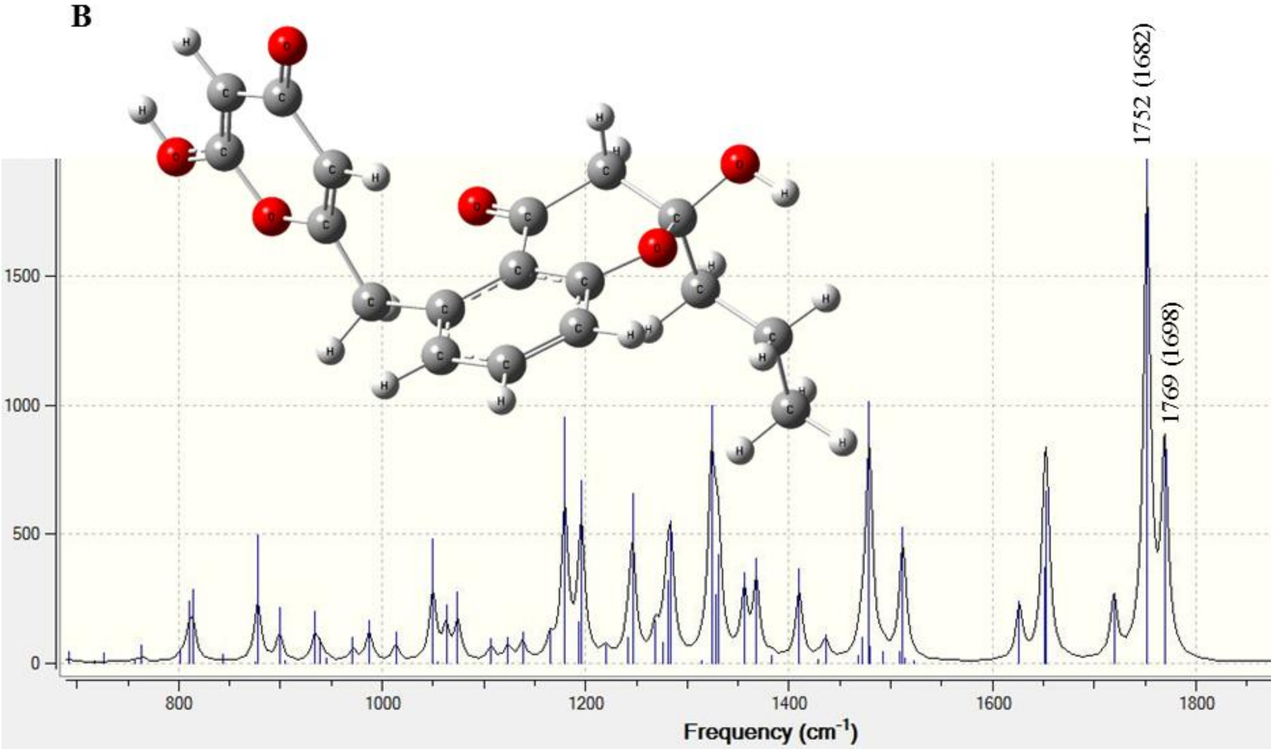

Figure S17. Experimental FT-IR spectrum of compound **13**.

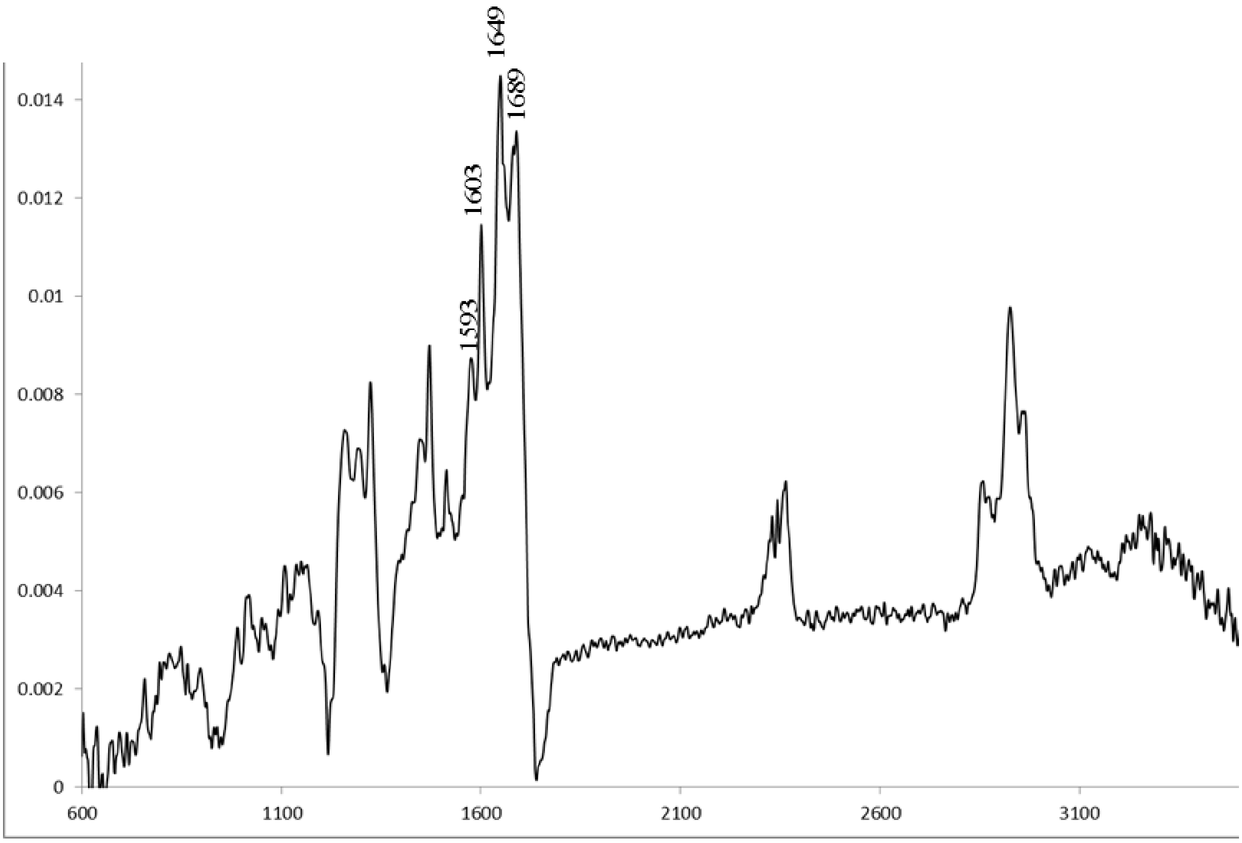

Table S1. Antibacterial activities at 100 μL/well of compounds **9**, **11** and **13**.

**Compounds**	**Inhibition Zone ± SD (mm)**				
	***E. coli* ATCC 25922**	***S. aureus* ATCC 25923**	***MRSA* ATCC 43300**	***B. subtilis* ATCC 6633**	***P. aeruginosa* ATCC 27853**
**9**	-	-	08.34 ± 1.04	-	11.07 ± 1.23
**11**	08.08 ± 0.41	-	08.27 ± 0.38	-	07.61 ± 0.71
**13**	12.44 ± 0.84	-	15.033 ± 0.57	-	13.56 ± 0.84
Vancomycin (30 μg/disc)	ND	20.05 ± 0.60	ND	ND	ND
Erythromycin(15 μg/disc)	ND	25.26 ± 0.64	14.63 ± 0.27	ND	ND
Gentamicin (10 μg/disc)	23.37 ± 0.5	25.78 ± 0.30	ND	ND	18.88 ± 0.27

-: No activity observed; ND: not determined.
